# Biofuel production: an odyssey from metabolic engineering to fermentation scale-up

**DOI:** 10.3389/fmicb.2014.00344

**Published:** 2014-07-09

**Authors:** Whitney Hollinshead, Lian He, Yinjie J. Tang

**Affiliations:** Department of Energy, Environmental and Chemical Engineering, Washington UniversitySt. Louis, MO, USA

**Keywords:** ATP maintenance, hydrodynamics, metabolic flux analysis, proteomics, synthetic biology

## Abstract

Metabolic engineering has developed microbial cell factories that can convert renewable carbon sources into biofuels. Current molecular biology tools can efficiently alter enzyme levels to redirect carbon fluxes toward biofuel production, but low product yield and titer in large bioreactors prevent the fulfillment of cheap biofuels. There are three major roadblocks preventing economical biofuel production. First, carbon fluxes from the substrate dissipate into a complex metabolic network. Besides the desired product, microbial hosts direct carbon flux to synthesize biomass, overflow metabolites, and heterologous enzymes. Second, microbial hosts need to oxidize a large portion of the substrate to generate both ATP and NAD(P)H to power biofuel synthesis. High cell maintenance, triggered by the metabolic burdens from genetic modifications, can significantly affect the ATP supply. Thereby, fermentation of advanced biofuels (such as biodiesel and hydrocarbons) often requires aerobic respiration to resolve the ATP shortage. Third, mass transfer limitations in large bioreactors create heterogeneous growth conditions and micro-environmental fluctuations (such as suboptimal O_2_ level and pH) that induce metabolic stresses and genetic instability. To overcome these limitations, fermentation engineering should merge with systems metabolic engineering. Modern fermentation engineers need to adopt new metabolic flux analysis tools that integrate kinetics, hydrodynamics, and ^13^C-proteomics, to reveal the dynamic physiologies of the microbial host under large bioreactor conditions. Based on metabolic analyses, fermentation engineers may employ rational pathway modifications, synthetic biology circuits, and bioreactor control algorithms to optimize large-scale biofuel production.

## Microbial factories for biofuel production

**Figure 1:**
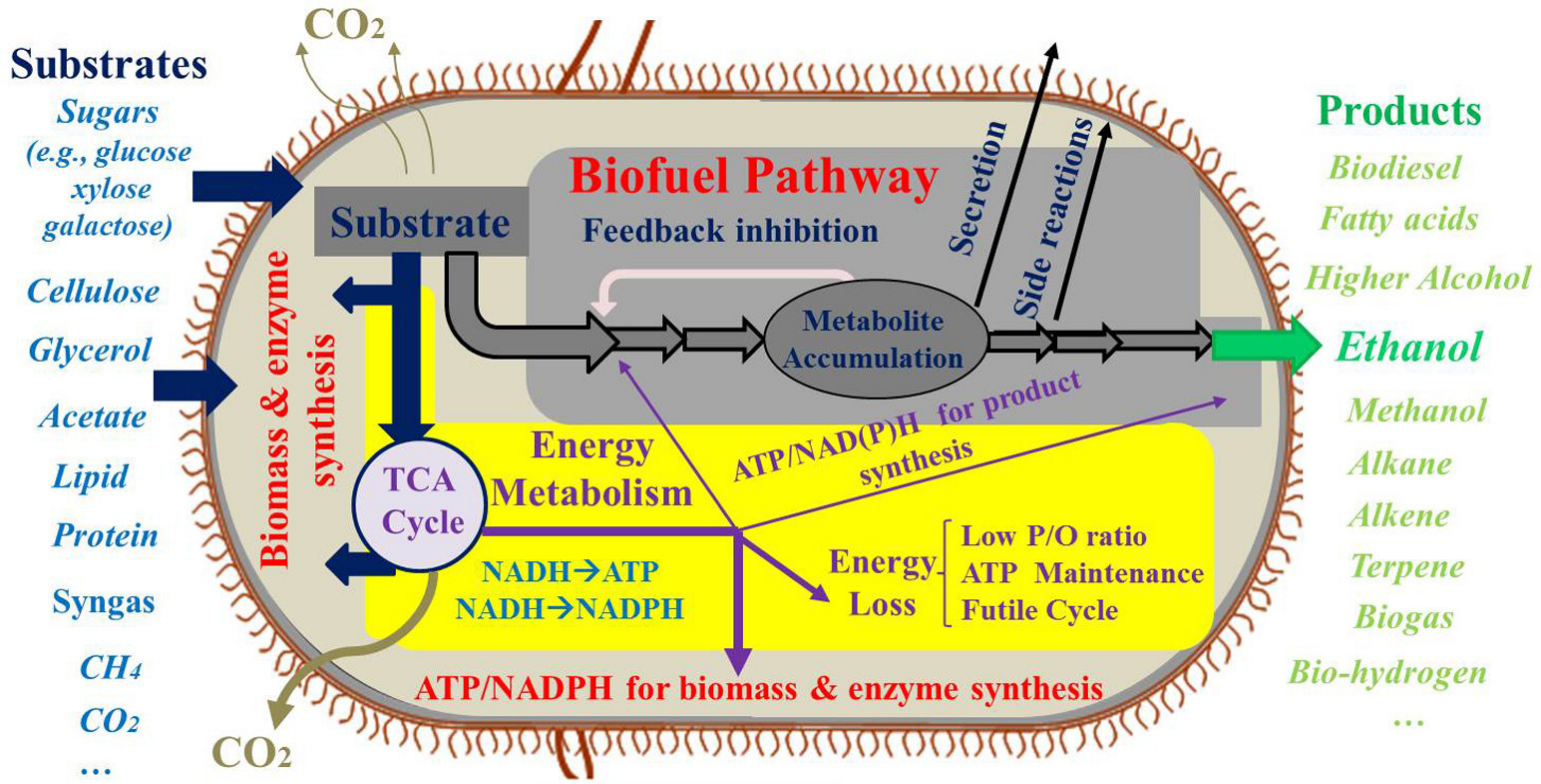
**Carbon and energy limitation for biosynthesis**.

Extensive research has been performed on the microbial production of biofuels using renewable feedstocks (Figure 1). Carbohydrates, the major carbon sources for biofuel production, can be obtained from either food crops or lignocellulosic biomass. Glycerol, lactate, acetate, and syngas are also used as feedstocks for biofuel synthesis. Moreover, photo-biorefineries are being developed to turn light energy and CO_2_ into useful chemicals (Lindberg et al., 2010; Lan and Liao, 2012; Oliver et al., 2013). Recently, the Department of Energy has started initiatives to target methane as a cheap resource for “gas-to-liquids” bioconversion in the hope of surpassing Fischer-Tropsch process for liquid fuel production (Conrado and Gonzalez, 2014). Despite the numerous feedstocks that are proposed for biofuel fermentation, production of cheap biofuel has not yet been realized.

Ethanol fermentation by yeast is the most developed biofuel process, but low combustion energy and high purification costs prevent the wide use of ethanol as an economical fuel. Thereby, researchers have engineered microbes to produce new fuels. Advanced biofuel examples include higher alcohols via the keto-acid and the Ehrlich pathway (Atsumi et al., 2008), terpene-based fuels (e.g., isopentenol) from the mevalonate pathway (Withers et al., 2007), fatty acid ethyl esters (Kalscheuer et al., 2006; Steen et al., 2010) and alkanes from fatty acid biosynthesis pathways (Choi and Lee, 2013). These biofuel pathways have been extensively reviewed (Rude and Schirmer, 2009; Peralta-Yahya et al., 2012). Despite the development of these diverse biofuel producers, it is still challenging to commercialize biofuel processes due to the poor microbial productivity in large bioreactors and the low profit margins of biofuels (Zhang, 2009; Lamonica, 2014). As a result, many biotechnology companies have shifted their focus away from advanced biofuels to products with higher commercial value. This review discusses both the metabolic and bioprocess limitations in the scale-up of these biofuel processes and emphasizes the need to integrate systems biology, synthetic biology, and fermentation engineering to optimize metabolic performance in large bioreactors.

## Metabolic engineering approaches for biofuel synthesis

The microbial host's metabolism consists of thousands of chemical reactions that control the carbon and energy [e.g., ATP & NAD(P)H] metabolism. The desired biosynthetic pathway for advanced biofuels often requires multiple enzymatic steps. Current molecular biology techniques can effectively alter enzyme levels to increase the flux toward biofuel synthesis. Common strategies include choice of plasmids and its copy numbers, promoter engineering, codon optimization, synthetic scaffolds, directed evolution/modification of key enzymes, improvement of ribosome binding sites, and knockout/knockdown of competitive pathways (Dueber et al., 2009; Carneiro et al., 2013; Nowroozi et al., 2014). New genetic techniques, such as RNA Interference, CRISPRs or TALENs, offer new capabilities to edit microbial metabolisms (Pratt and MacRae, 2009; Jiang et al., 2013; Sun and Zhao, 2013).

To improve the carbon flux to the final products, metabolic engineering often applies two strategies. The first strategy is the “push-pull-block,” used to increase the flux toward the biofuel synthesis pathway (Atsumi et al., 2010a; Kind et al., 2013). For example, a threonine-overproducing *E. coli* strain was engineered to produce 1-propanol via the keto-acid pathway by the “push-pull-block” strategy (Choi et al., 2012): (1) “Pull”—introduce a heterologous feedback resistant threonine dehydratase, (2) “Block”—remove competing metabolic pathways, and (3) “Push”—overexpress acetate kinase and other enzymes in the citramalate pathway to increase carbon flux into the propanol pathway. The second metabolic engineering strategy is to design an alternative biosynthesis pathway that can reduce the loss of carbon by unwanted byproducts. For example, a non-oxidative glycolytic cycle in *E. coli* has been developed to achieve the complete carbon conversion of sugar into acetyl-CoA (Bogorad et al., 2013). Although these metabolic engineering strategies are effective in increasing the carbon flux toward the desired product, metabolic engineers cannot easily create “biofuel super bugs”. Extensive genetic modifications often increase metabolic burdens on the host and thus further interfere with cell growth and product synthesis (Colletti et al., 2011; Poust et al., 2014). For example, high copy number plasmids or strong promoter can place a heavy burden on the cell's growth and negatively affect productivity (Carrier et al., 1998; Jones et al., 2000). Moreover, host cells may incorrectly express or misfold heterologous enzymes, reducing their activities. Low temperature fermentation may be required to ensure the functions of these heterologous enzymes (Chang et al., 2007). In addition, pathway engineering may cause metabolic imbalances and waste product secretions.

An emerging field, synthetic biology, aims to design and construct new biological systems to enhance the capability of engineered microbes (Nielsen et al., 2014). Synthetic biology has been developing genetic circuits that can precisely regulate gene expression in the presence or absence of chemical and environmental inputs (Khalil and Collins, 2010). These synthetic biological devices have been reviewed recently (Way et al., 2014), which include such devices as a toggle switch (two repressors turn each other off), trigger-memory system, and genetic oscillators. Synthetic biology tools have started to be used by metabolic engineers to manipulate fluxes toward biosynthesis pathways at different fermentation stages. For example, a recent study engineered a toggle switch into *E. coli* that could turn off the TCA cycle and redirect flux toward isopropanol (Soma et al., 2014). Among the synthetic biology tools (Neupert et al., 2008; Topp et al., 2010; Gorochowski et al., 2013), biosensor-regulator systems have particular value for their potential to control a microbial host metabolism according to environmental changes, and thus improve the productivity of microbial hosts (Zhang et al., 2012).

## Metabolic dilemma: carbon yield vs. energy efficiency

Current research often focuses on the improvement of carbon fluxes toward the final product. However, the high demand of energy and reducing equivalents during biofuel synthesis is another important obstacle. First, polymerization of protein and DNA/RNA requires large amounts of ATP (39.1 mmol ATP/g protein; 7.4 mmol ATP/g RNA; and 11.0 mmol ATP/g DNA) (Stephanopoulos et al., 1998). Production of biomass, enzymes for biofuel synthesis, plasmids/mRNA, or synthetic scaffolds consumes not only carbon building blocks, but also energy molecules. Second, large amounts of ATP need to be consumed to support cell maintenance processes including energy spilling, microbial motility, cell component repair, and re-synthesis of macromolecules (Hoehler and Jorgensen, 2013). Third, synthesis of biofuel molecules needs ATP and NAD(P)H. For example, fatty acid production requires 7 ATP and 14 NADPH to convert acetyl-CoA molecules into one fatty acid (Palmitate, C16:0). The carbon oxidation pathways (such as TCA cycle and oxidative pentose phosphate pathway), oxidative phosphorylation, and transhydrogenase reactions are required to generate sufficient NADPH and ATP for fatty acid synthesis (He et al., 2014; Varman et al., 2014).

Theoretically, 38 ATP molecules are produced from one glucose molecule. Among the 38 ATP molecules, glycolysis and TCA pathways only contribute to 4 ATP, and the remaining 34 ATP are obtained from oxidative phosphorylation, assuming the maximum P/O ratio (1 NADH → 3 ATP and FADH_2_ → 2 ATP) (Shuler and Kargi, 2002). Under anaerobic conditions, the energy metabolism is inefficient and cells often secrete acetate to overcome the ATP shortage. If the biofuel synthesis requires large amounts of ATP, oxidative phosphorylation becomes a key source for satisfying the ATP demand (i.e., use of aerobic respiration to generate the needed ATP). In addition to the high ATP demand imposed by the biofuel synthesis pathway, metabolic flux analysis studies have revealed that the overexpression of biosynthesis pathways significantly increases ATP maintenance expenditure (Ow et al., 2009), and the metabolic burden in engineered microbial hosts further causes poor respiration efficiency (e.g., P/O ratio = 1.3) (Varma and Palsson, 1994; Sauer and Bailey, 1999). If the hosts suffer from severe ATP limitations, efforts to reduce carbon losses or to increase carbon availability to biofuel synthesis will be futile. In this case, the metabolic bottleneck may shift from carbon limitation to energy limitation (insufficient energy molecules to power biofuel synthesis). Many metabolic engineering approaches to improve carbon efficiency are effective in redirecting carbon fluxes to biofuel in low productivity strains (yield far below theoretical value), but these strategies cannot raise product yields close to stoichiometric predictions if the engineered metabolism is unable to satisfy the overall ATP and NAD(P)H requirements by the microbial hosts. The priorities toward high carbon yield and energy efficiency have to be carefully balanced during strain development of biofuel producers.

## Scale-up fermentation obstacles: metabolic stresses under suboptimal culture conditions

Fermentation engineering emerged in the early 1940s driven by the mass production of penicillin. Since then, engineers have scaled up fermentation of commodity products such as 1,3-propanediol (Nakamura and Whited, 2003) and amino acids (Hermann, 2003). Currently, Gevo and Butamax are commercializing isobutanol production from engineered yeast (Nielsen et al., 2014). Industrial isobutanol fermentation is one of the most promising biofuel fermentations as: (1) yeast is a robust industrial host that has a natural tolerance to alcohols; and (2) *in situ* removal techniques such as gas-stripping have alleviated product toxicity during the fermentation process (Baez et al., 2011). However, large-scale fermentations of other fuels (such as biodiesels) are still underdeveloped.

Maintaining the optimal growth environments in large bioreactors is difficult. In industrial bioreactors (on the scale of 100 m^3^), the poor mixing/aeration can cause temperature and pH fluctuations, O_2_ limitations, substrate/product inhibitions, and accumulations of wastes (such as acetate) (Enfors et al., 2001). The heterogeneous conditions can increase cell stress and ATP maintenance. If gaseous substrates (such as CO_2_, syngas or CH_4_) are used for fermentation, gas-liquid mass transfer resistances pose another serious challenge as the gaseous substrates must diffuse across the gas-liquid interface (Blanch and Clark, 1997). In addition, large-scale bioreactors (e.g., a fed-batch bubble column reactor) feed the substrate from the top and aeration from the bottom, creating opposite substrate and O_2_ gradients (i.e., O_2_ limitation at the top and substrate limitation at the bottom). This has been reported to cause increased production of waste products, such as formate, lactate, and succinate (Bylund et al., 1998, 2000). Moreover, cell factories synthesizing biofuels are subjected to metabolic burdens due to the drainage of both metabolic precursors and energy for the replication of plasmids, and biosynthesis of heterologous enzymes and products (Carneiro et al., 2013). The suboptimal growth conditions in large bioreactors tend to intensify stress responses, induce metabolic shifts, and alter cell genetic stability. Altogether, most of the engineered “super bugs” struggle to move beyond lab settings.

## ^13^C-MFA: an indispensable tool for scale-up fermentation engineering

**Figure 2:**
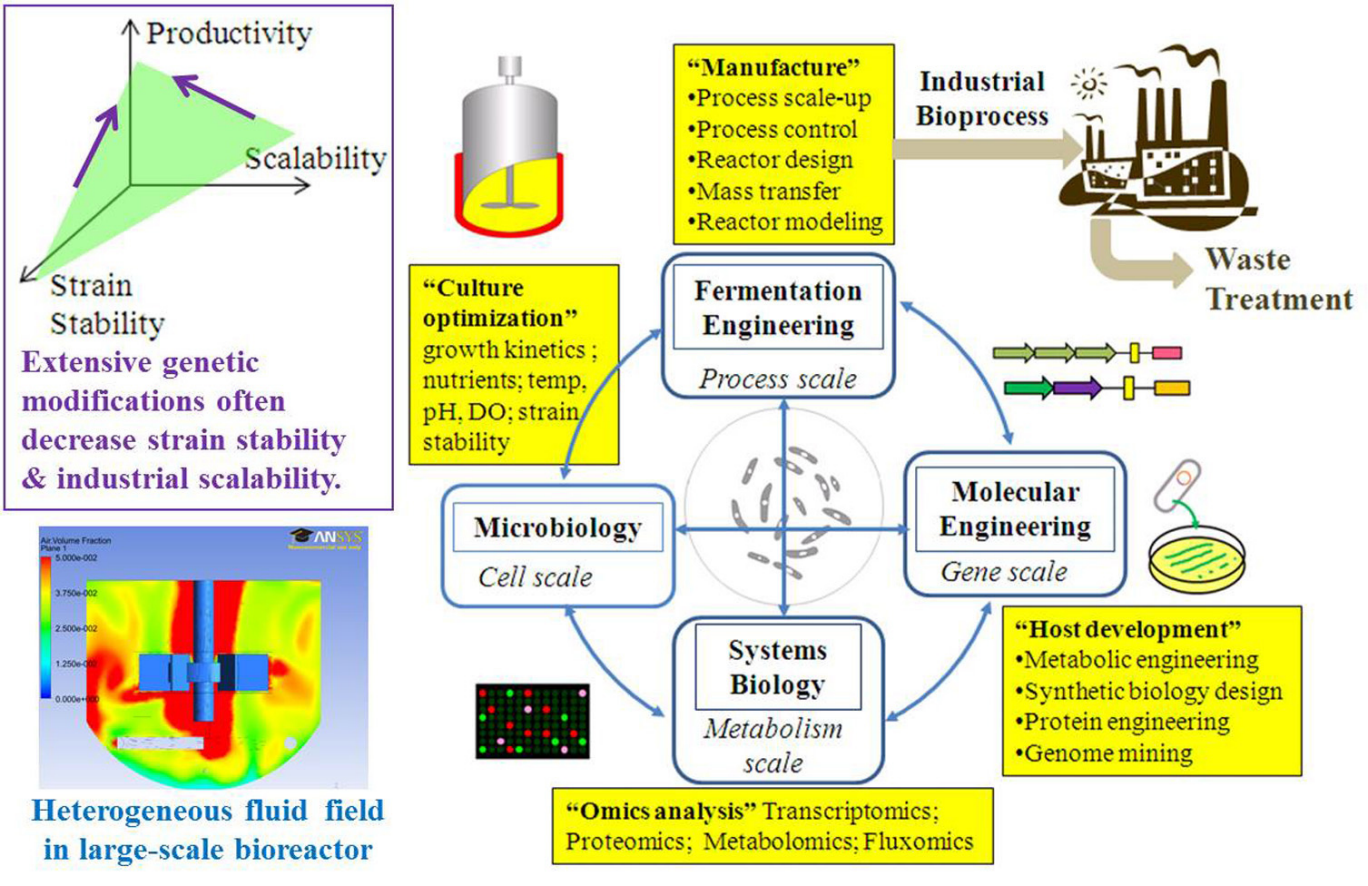
**The design-engineering-analysis cycle for scale-up of biofuel fermentation**.

Systems biology can characterize biofuel producers and provide guidelines for rational metabolic engineering and optimal fermentations (Figure 2) (Carneiro et al., 2013). “Omics” (transcriptomics, proteomics, and metabolomics) approaches can identify useful mutations, discover gene regulations and enzyme functions, and measure the metabolite pools in response to environmental fluctuations (Pham et al., 2006; Atsumi et al., 2010b; Redding-Johanson et al., 2011). Although genome-wide analyses and computational modeling have provided knowledge for the metabolic engineering of microbial hosts (Park et al., 2008), it is not straightforward to correlate “omics” to actual enzyme functions due to complex post-transcriptional regulations (such as allosteric regulation) (Chubukov et al., 2013). For example, increases in mRNA levels may not lead to a corresponding increase in protein levels, while enzyme levels can remain constant despite frequent changes in carbon fluxes (Gygi et al., 1999). In addition, the existence of isoenzymes and poor enzyme specificity contribute to high metabolome diversity and lead to difficulties in metabolomic studies (Schwab, 2003). To overcome such problems, ^13^C-MFA (metabolic flux analysis) is used to directly measure enzymatic reaction rates. Compared to other omics studies, ^13^C-MFA can provide insights into cell's physiology during large-scale fermentations. ^13^C-MFA uses metabolic reaction stoichiometry and carbon-labeling experiments to precisely estimate metabolite turnover rates (Sauer, 2006; Tang et al., 2009). ^13^C-MFA in combinations with other *in silico* metabolic models (flux balance analysis) (Edwards et al., 2001; Orth et al., 2010) can predict biosynthesis yield, delineate functional pathways, calculate the actual fluxes throughout the metabolic network, validate the function of genetic circuits, and identify metabolic engineering targets. Integration of flux analysis with transcriptomics and proteomics can provide a comprehensive understanding of the genetic regulations of intracellular activities (Zhang et al., 2010; Yoon et al., 2012; Arakawa and Tomita, 2013; Liu et al., 2013).

During the fermentation process, metabolic shifts and genetic mutations are common, which can create subpopulations in the cultures (biofuel producers vs. mutants after loss of biofuel production). Sauer group proposed a “reporter protein”-based ^13^C-MFA to probe the metabolism of subpopulations (Rühl et al., 2011). This reporter protein, synthesized by a particular subgroup, stores the ^13^C-labeling information of that subgroup which allows ^13^C-MFA to identify the different metabolic flux phenotypes. This proof-of-concept approach was tested using engineered *E. coli* (Shaikh et al., 2008; Rühl et al., 2011). Based on the labeling information from hydrolysates (i.e., amino acids) of the green fluorescent protein (GFP), ^13^C-MFA was able to probe the subpopulation metabolism (GFP producers) during *E. coli* fermentation. Recently, proteomic analysis of ^13^C-peptide labeling has also been developed to assist metabolic flux analysis of heterogeneous microbial systems (Mandy et al., 2014). The “reporter proteomics” associated with a specific time of protein expression or a unique species may decipher subpopulation physiologies and its dynamic changes in microbial consortia. These studies have paved the way to analyze temporal and spatial microbial phenotypes during a large-scale heterogeneous fermentation.

In addition, ^13^C-MFA may integrate with microbial kinetics to reveal the changes of cell metabolism throughout its cultivation process. For example, Sauer group have utilized such a dynamic ^13^C-MFA approach in a fed-batch fermentation to monitor the dynamic changes in intracellular fluxes during the different growth stages. Their results revealed that a riboflavin-producing bacterium's physiology shifted from an overflow metabolism to an exclusively maintenance metabolism at the late fermentation stage (Rühl et al., 2010). To determine microbial responses to industrial bioreactor configurations and cultivation heterogeneity due to mass transfer limitations, even more novel ^13^C-MFA approaches are required. For example, metabolic flux analysis in combination with hydrodynamics would provide important insights into cell physiologies at different locations inside of a large bioreactor. However, to the best of our knowledge, such metabolic flux analysis techniques have not been fully developed.

## Modern fermentation engineering: an integrated approach for biofuel fermentation manufacturing

The promise of cheap biofuels has yet to be fulfilled. Currently, alcohols (though of a lower energy density) are more promising biofuels than biodiesel/hydrocarbons, as alcohol synthesis pathways are less dependent on the ATP supply and alcohol fermentation can be conducted under anaerobic conditions. Production of biodiesel and hydrocarbon require large amounts of ATP, and their processes are usually restricted to aerobic conditions and their productivities are highly sensitive to the P/O ratio (He et al., 2014; Varman et al., 2014). To date, researchers are still unable to create “super bugs” that have both efficient carbon metabolism and frugal energy usage for economical synthesis of advanced biofuels. Thereby, modern fermentation engineering needs to be merged with advanced metabolic engineering to employ the following strategies.

First, it is important to select and develop a proper biofuel chassis with an efficient energy and carbon metabolism. Yeast (e.g., *Saccharomyces cerevisiae*) is naturally tolerant to alcohols and acetate, and it is a robust workhorse for industrial alcohol fermentations. However, the energy metabolism of yeast has a lower net ATP production as additional ATP is consumed during the transport of equivalents of NADH from the cytoplasm into the mitochondria (Shuler and Kargi, 2002). In addition, the mitochondrial metabolite transport processes may limit the enzyme-substrate accessibility during biofuel synthesis. Thereby, compartmentalization of biosynthetic pathways in yeast's mitochondria is required to improve local enzyme concentrations and microenvironments for biofuel synthesis (Avalos et al., 2013). On the other hand, a bacterial chassis, *E. coli*, has faster growth and product synthesis rates, pathways that are easier to modify, and a broader capability to co-utilize carbon sources (including xylose). *E. coli* has already been engineered to produce higher alcohols, biodiesels and other hydrocarbons that demand large amounts of energy molecules. Another promising chassis is microalgae (i.e., cyanobacteria). Although most studies emphasize their photoautotrophic features, photomixotrophic fermentation has two great advantages for industrial use. (1) Photo-fermentations of organic substrates (sugars) can reach high carbon yields of both biofuel and biomass due to mixotrophic CO_2_ fixation and light energy harvesting to generate ATP and NADPH (You et al., 2014). (2) Photo-fermentation by cyanobacteria has no overflow byproducts and is less susceptible to CO_2_/light limitations during the bioprocess scale-up. Another possible direction, non-model species, such as thermophiles, are also promising for cheap biofuel production because such species are particularity suited for consolidated bioprocesses (Lin et al., 2014). Finally, it might be possible to construct a new microbial chassis using a synthetic genome in the near future, which may be developed to contain all the advantageous features of the other microbial hosts. These synthetic or non-model microbes may eventually fulfill the promise of cheap biofuels.

Second, the loss of carbon yield due to ATP/NAD(P)H limitations is often severe in engineered microbial hosts. Therefore, modern fermentation engineering should focus on reducing the metabolic burden and enhancing the prosperity of the energy metabolism. There are several effective strategies to improve NAD(P)H and ATP availability. (1) Adding nutrient sources (such as yeast extract) into cell culture; (2) overexpressing NADH dehydrogenase to enhance the respiration efficiency (Calhoun et al., 1993); (3) using engineered enzymes to balance NADH/NADPH generation and consumption (Berrıos-Rivera et al., 2002; Javidpour et al., 2014); (4) employing *in situ* product recovery/separation to avoid culture inhibitions (Baez et al., 2011); and (5) maintaining the optimal cultivation conditions (such as dissolved O_2_, substrate concentrations, etc.).

Third, traditional fermentation engineering aims to understand bioreactor rheology and bio-reaction kinetics, but there should be a deeper understanding of the microbial host in large bioreactors. Fermentation engineers often study process control parameters such as mixing quality, oxygen/nutrient supply, reactor geometry, impeller selection, aeration rate, heat transfer, power-volume ratio, and the necessary utility operation costs (Delvigne et al., 2005). For example, fermentation engineers have designed fed batch algorithms to avoid acetate secretion by *E. coli* (Shuler and Kargi, 2002). However, future fermentation engineering should also combine process analyses (including computational fluid dynamics, structured kinetic models, and scale-up simulations) with advanced metabolic flux analysis to capture the cell's behavior and dynamics under the vessel's specific conditions and provide guidelines for process design and control. Systems analysis has been successfully used as a fermentation engineering technique to reveal metabolic bottlenecks, design optimal culture medium, and monitor physiological performance under different bioreactor conditions (Becker et al., 2013; Posch et al., 2013). For example, researchers from Genentech have integrated bioprocess models with metabolic flux analysis to study *E. coli* metabolism in a 1000 L bioreactor for the production of a recombinant therapeutic protein. The integrated modeling approach can be used to optimize process variables and media compositions to unlock the optimal cell metabolism for efficient biosynthesis (Meadows et al., 2010). As expected, future fermentation engineering can significantly benefit from combining metabolic flux analysis with mass transport and hydrodynamics to understand cell physiologies in heterogeneous/dynamic bioreactor environments and identify the bioreactor stress factors.

**Figure 3:**
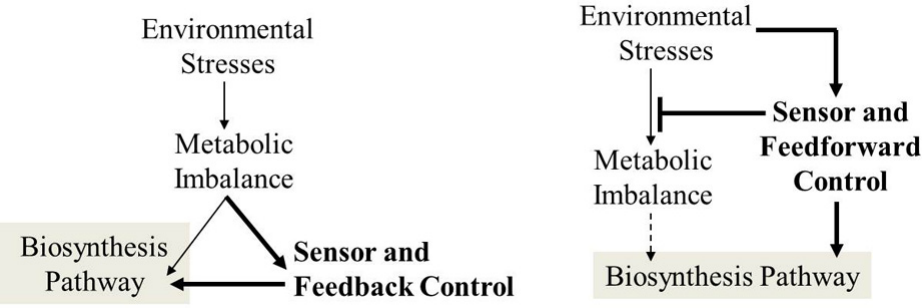
**Application of feedback and feedforward sensor-regulator systems to improve cell responses under suboptimal conditions**.

Fourth, synthetic biology offers new opportunities for fermentation scale-up. Introducing synthetic dynamic control systems can prevent the biosynthesis of unnecessary RNAs/proteins/metabolites, increase the efficiencies of energy and carbon usage, and allow a host to adjust its metabolic flux to minimize “maintenance loss” (Zhang et al., 2012). Metabolic pathways can be regulated via transcription factors that quickly respond to shifts in metabolite levels, and thus fluxes in the biosynthetic pathway could be controlled on the time scale of minutes (Holtz and Keasling, 2010). To build dynamic regulatory capability in a microbial host, biosensor-regulators can be used to promote or repress a biofuel pathway or substrate uptake according to its growth conditions (e.g., quorum sensing) or metabolite concentrations (Dunlop et al., 2010). Recently, a dynamic feedback control system was developed that enabled the host to sense metabolic changes by detecting the toxic intermediate's concentration (e.g., malonyl-CoA or acyl-CoA), and thereby controlling the expression of biodiesel synthesis genes (Zhang et al., 2012). Such a feedback control allows cells to maintain the concentration of the precursor and intermediate at desirable levels, and thus both genetic stability and productivity in the new strains were improved. In addition, biosensors of feedforward control, will also be beneficial in the scale-up of fermentations, because they allow cells to respond to environmental changes before a deleterious impact on cell metabolism. For example, O_2_ limitation leads to acetate secretion and glucose over-consumption during aerobic fermentations. O_2_ sensitive promoters (Salmon et al., 2003) can be used to control glucose transporter genes and “shut down” glucose uptake when the cells move to O_2_ limiting zones in a large bioreactor (Figure 3). Although bio-sensing regulatory systems can optimize biomass growth and product synthesis at different growth phases/conditions in laboratory settings, applicability of synthetic biology in scale-up fermentation is still unclear. Recently, the stability of genetic circuits (AND and NOR gate) were evaluated under simulated industrial fermentation conditions, and the circuit's performance (AND gate) deteriorated with increased culture volume (Moser et al., 2012). Thereby, it is necessary for fermentation engineers to test and improve synthetic biology circuits under dynamic and heterogeneous cultivation conditions.

## Summary

Optimization of biofuel fermentation at the industrial scale is difficult and costly. Biofuel fermentation engineering should integrate with metabolic engineering to tune the expression of multiple heterologous genes, improve the energy metabolism (high P/O ratio and low cell maintenance), and construct sensor-regulator systems to improve cell productivity in industrial bioreactors. Fermentation engineers should have a comprehensive understanding of both the macroscopic (e.g., oxygen level, mixing, and bioreactor controls) and microscopic (intracellular fluxes) parameters, and thus fill the gaps between laboratory studies and industrial applications. “Scale-down” experiments (large-bioreactor fermentation simulated on small scales) with metabolic flux analysis can be routinely used to diagnose the engineered metabolism, verify synthetic biology circuits, and design optimal fermentation strategies (Figure 2). Most importantly, this broad-scope metabolic knowledge would allow companies to select and focus on “promising” microbial factories with high chances of success.

### Conflict of interest statement

The authors declare that the research was conducted in the absence of any commercial or financial relationships that could be construed as a potential conflict of interest.
